# An Enriched Environment Promotes Motor Function through Neuroprotection after Cerebral Ischemia

**DOI:** 10.1155/2023/4143633

**Published:** 2023-02-08

**Authors:** Liang-Feng Shi, Chuan-Jie Wang, Ke-Wei Yu, Jun-Fa Wu, Qi-Qi Zhang

**Affiliations:** ^1^Department of Endocrine Medicine, Jinshan Hospital, Fudan University, Shanghai, China; ^2^Department of Rehabilitation Medicine, Jinshan Hospital, Fudan University, Shanghai, China; ^3^Department of Rehabilitation Medicine, Huashan Hospital, Fudan University, Shanghai, China

## Abstract

Stroke seriously affects human health. Many studies have shown that enriched environment (EE) can promote functional recovery after stroke, but the intrinsic mechanisms remain unclear. In order to study the internal mechanisms of EE involved in functional recovery after ischemic stroke and which mechanism plays a leading role in the recovery of limb function after cerebral infarction, key proteins potentially involved in neuronal protection and synaptic remodeling in the ischemic penumbra have been investigated. In this study, adult C57BL/6 mice after permanent middle cerebral artery occlusion (pMCAO) were assigned to the EE and standard housing (SH) groups 3 days after operation. The EE house was spacious that contained a large variety of small toys; the SH was a normal sized cage. Sham-operated mice without artery occlusion were housed under standard conditions and were fed a normal diet. On days 3, 7, 14, and 21, postoperative motor functional recovery was tested using the modified neurological severity score (mNSS) and the Rotarod test. The expression of B-cell lymphoma-2 (Bcl-2), Bcl-2-associated X protein (Bax), growth-associated protein-43 (GAP-43), and synaptophysin (SYN) was examined by western blotting and immunofluorescence staining. The motor functional recovery (based on the mNSS and Rotarod test 3, 7, 14, and 21 days post operation) of mice in the EE group improved significantly compared to the SH group. The expression of GAP-43 and SYN and the ratio of Bcl-2/Bax were all upregulated in the EE group compared to the SH group. In addition, we also explored the relationship between neuronal protection and synaptic remodeling in the EE-mediated recovery of limb function after cerebral infarction by correlation analysis. Correlation analysis showed that compared with the increase of Bcl-2/Bax ratio, the increased expression of GAP-43 and SYN was more closely related to the recovery of limb function in ischemic mice. These data support the hypothesis that EE can promote the process of improvement of limb dysfunction induced by ischemic stroke, and this behavior restoration may, via promoting neuroprotection in the ischemic penumbra, be dependent on the regulation of the expression of GAP-43, SYN, Bcl-2, and Bax. A limitation of the study was that we only observed several representative key indicators of synaptic remodeling and neuronal apoptosis, without an in-depth study of the potential mechanisms involved.

## 1. Introduction

Stroke is still one of the main diseases leading to human disability in the world [1]. A large number of neuronal cell deaths and synaptic activity disorders in the peri-infarct area are the main causes of limb dysfunction after ischemic stroke [2, 3]. Therefore, improving the neuronal function of the ischemic penumbra by promoting neuroprotection is considered a promising therapeutic strategy to treat ischemia.

An enriched environment (EE), as one of the most effective ways to improve neural plasticity, has received much attention of late [4, 5]. EE generally refers to a large cage which contains many tunnels, toys, small houses, and so on, in order to increase social interaction and physical exercise. Previous studies have indicated that an EE involves many different mechanisms that contribute to neural plasticity, which is the foundation of neural repair after brain injury [6, 7], but the internal mechanisms involved still require in-depth study.

Elongation of axons has been one of the most important observations made during synaptic plasticity studies. Growth-associated protein-43 (GAP-43) plays an essential role in neuronal plasticity and is commonly used as a biological indicator of neuronal development and regeneration [8]. Synaptophysin (SYN) is an important marker of synaptic remodeling and is widely expressed in the presynaptic vesicles of neurons [9]. Previous research has found that SYN expression was significantly downregulated after ischemic stroke [10]. Our previous study found that an EE can reverse the reduction of SYN in the hippocampal CA1 region, a finding that is most likely related to the improvement of cognitive function after cerebral infarction [11]. Therefore, GAP-43 and SYN are clearly two very important indicators of synaptic remodeling.

Neuronal apoptosis is regulated by various members of the Bcl-2 family, with Bcl-2 and Bax being two important proteins that play a role in apoptosis [12]. The ratio of Bcl-2/Bax can be used to determine the degree of apoptosis of neurons. Reducing neuronal apoptosis by upregulation of the ratio of Bcl-2/Bax is conducive to the recovery of neural functions after cerebral infarction.

Focal cerebral ischemia can activate apoptosis and synaptic dysfunction leading to serious impairment of neural activity. Existing studies suggest that EE can reduce neuronal apoptosis and synaptic dysfunction after cerebral infarction. However, there are few reports on whether EE intervention can improve neuronal apoptosis and promote synaptic remodeling at the same time. And few studies have reported that the neuronal protection or synaptic remodeling plays a leading role in the EE-mediated recovery of limb function after cerebral infarction. In the present study, the motor functional recovery was tested using the modified neurological severity score (mNSS) and the Rotarod test. And we observed several representative key indicators of synaptic remodeling (GAP-43 and SYN) and neuronal apoptosis (Bcl-2 and Bax) by western blotting and immunofluorescence staining. In order to expose the neuronal protection or synaptic remodeling that plays a leading role in the EE-mediated recovery of limb function after cerebral infarction, the correlation of the motor functional recovery outcomes with GAP-43, SYN, and Bcl-2/Bax was analyzed using Spearman's correlation coefficient.

## 2. Materials and Methods

### 2.1. Animals

Sixty healthy, specific pathogen-free (SPF) C57/BL6 male mice (Zhejiang Vital River Laboratory Animal Technology Co., Ltd., Zhejiang, China), aged 3 months old and weighing between 27 and 30 g, were used in the experiments. All animal experiment protocols were approved by the Institutional Animal Care and Use Committee of Fudan University, approval No. 2020 Huashan Hospital JS-163.

**Figure 1:**
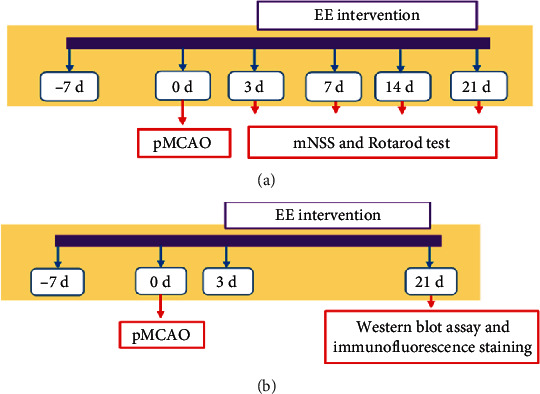
Schematic of the experimental design. (a) mNSS and Rotarod tests were conducted on days 3, 7, 14, and 21 after pMCAO. (b) Brain samples analyzed by western blotting and immunofluorescence staining were collected on day 21 after pMCAO.

Of the 60 mice, 16 were randomly selected as the sham group, and 44 underwent a left permanent middle cerebral artery occlusion (pMCAO) operation. The 44 mice were evaluated after the operation, and 32 of them qualified for inclusion in the study. Mice meeting the inclusion criteria were randomly assigned to an EE group (*n* = 16) or a standard housing (SH) group (*n* = 16) on day 3 post operation. The groups were raised in cages under an EE or SH, respectively. The sham group, without occlusion of the artery, was treated in cages under SH. A schematic of the experimental design is shown in Figure 1.

### 2.2. pMCAO Procedure

The animal surgery was performed as described in our previous study [11]. In brief, mice were anesthetized with 2.0% isoflurane, mixed with 30% oxygen and 68% nitrous oxide. Left arteries, including the common carotid artery, external carotid artery, and internal carotid artery, were dissected. A 6-0 silk suture was used to make a ligation of these arteries. A 4-0 nylon suture was inserted through an incision in the common carotid artery and stopped at the opening of the middle cerebral artery (MCA). A moorVMS-LDF2 laser Doppler flowmeter (Moor Instrument, Devon, UK) was used to check the blood flow to confirm that occlusion had been successfully achieved. The distance from the branch point to the opening of the MCA was circa 7 ± 0.5 cm, and an ipsilateral blood flow decrease of 65-70% compared to contralateral flow was regarded as successful occlusion. After the operation, each mouse was placed on an electric heating plate at 37°C until it recovered from the general anesthesia. All mice were then transferred to their respective feeding cages. The internal carotid artery of the sham mice was dissected, but without ligation being applied.

### 2.3. Animal Groups and the EE Paradigm

Three days post operation, in order to select mice that met the required experimental criteria, the limb functions of mice were evaluated on a beam walk as previously described [13]. Mice were evaluated on a 0–6 point scale as follows: walking freely on the balance beam = 0; 2 limbs grasp the beam but are weaker than normal = 1; 1 limb grabbed the balance beam and 1 falling off = 2; neither limb could grasp the balance beam, but the mouse did not fall off = 3; falling off the balance beam within 40–60 s = 4; falling off the balance beam within 20–40 s = 5; and falling off the balance beam immediately = 6. Mice with scores between 2 and 4 met the inclusion criteria and were randomly assigned to the EE or SH group. The sham-operated mice were maintained in standard cages under a standard environment.

**Figure 2:**
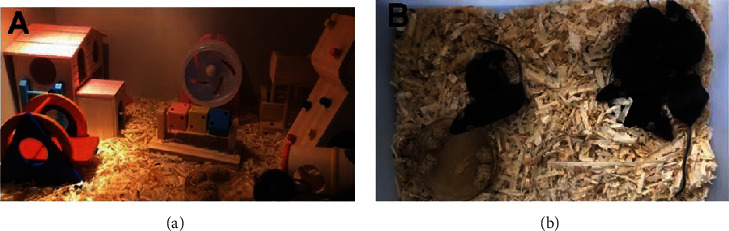
Environmental enrichment and standard housing conditions. (a) Enriched environment housing. (b) Standard housing.

The EE was a large wooden case (70 cm wide, 85 cm long, and 30 cm tall) (Figure 2(a)), containing various toys, including small houses, ladders, tubes, and running wheels. These toys were changed every 3 days with different shapes and colors. The SH and sham groups were kept in a normal sized cage (20 cm wide, 26 cm long, and 15 cm tall) (Figure 2(b)). Three days post operation, all mice had *ad libitum* access to water and food in their respective cages for 21 days.

### 2.4. Physical Function Assessment

The modified neurological severity score (mNSS) and Rotarod test were performed to assess the limb function recovery of all mice. The basic protocol of mNSS has been previously described [14]. Briefly, a scale from 0 to 14 was used to detect different levels of injury. Zero indicates normal, while 14 indicates the most severe injury. The Rotarod test was used to evaluate motion coordination ability. The detailed protocol of the Rotarod test is given in a previous study [15]. In brief, before pMCAO, mice were trained for 3 min at 5 rpm, 10 rpm, and 20 rpm for 3 days. After the training, the motor ability test of mice was repeated 3 times and the average value taken as the baseline value. The above 2 assessments were carried out on day 3, day 7, day 14, and day 21 after operation by two investigators who were blinded to the groupings. All tests were performed twice and the means calculated for use in statistical analyses.

### 2.5. Western Blot Assay

On postoperative day 21, the peri-infarcted cortex tissue was collected for western blot assay [10, 13]. The brain tissue around the infarct was purified into protein samples by the steps of lysis, homogenization, and purification. Equal amounts of the samples (20 *μ*L) were subjected to sodium dodecyl sulfate-poly-acrylamide (Bio-Rad, Berkeley, CA, USA) on 10% separation gel and 5% concentrated gel, and the samples were then transferred onto a 0.2 *μ*m nitrocellulose membrane (GE Healthcare Life Sciences, PA, USA). After being blocked for 1 h, the membranes were incubated with the following primary antibodies overnight: Bcl-2 (1 : 1,000), Bax (1 : 1,000), GAP-43 (1 : 1,500), SYN (1 : 1,500), and *β*-tubulin (1 : 1,000). All antibodies were purchased from Abcam (Cambridge, MA, USA). Membranes were then incubated with a secondary antibody (1 : 5,000; Abcam) at room temperature for 1 h. The optical densities of protein bands were quantified using a western blot imaging system (Bio-Rad, CA, USA), and tubulin was used as the relative target protein.

### 2.6. Immunofluorescence Staining

Mice were humanely killed 3 weeks after injury, and their brain samples were prepared as previously described [11]. Briefly, mice were perfused with 0.9% saline and 4% paraformaldehyde successively through the left ventricle after being deeply anesthetized with 5.0% isoflurane. Then, the brains were removed, fixed, dehydrated in methanol, and embedded in paraffin and 20 *μ*m slices cut using a freezing microtome. The immunofluorescence staining protocol has been previously described in detail [11]. In brief, the brain slices were fixed in methanol for 10 min at -20°C, and then, 10% BSA was used for blocking at room temperature for 1 h. Finally, the brain slices were incubated with the primary antibodies Bcl-2 and Bax (1 : 200), GAP-43 (1 : 200), and SYN (1 : 150) at 4°C overnight. Then, brain slices were incubated with secondary antibodies (anti-mouse IgG, 1 : 3,000) at room temperature after being thoroughly washed with PBS. All sections were viewed with a confocal microscope (Leica TCS SP2, Germany) using the same parameters. Four visual fields (400x) were randomly selected for each brain slice and digital images captured. All images were analyzed using Image-Pro Plus 6.0 (Bio-Rad, CA, USA).

### 2.7. Statistical Analysis

The results are presented as the mean ± SEM and were analyzed using SPSS ver. 19.0 (SPSS, Chicago, IL, USA). Data from the western blot assay, immunofluorescence staining, and mNSS and Rotarod tests were analyzed using one-way analysis of variance followed by Fisher's least significant difference post hoc test. The correlation of the motor functional recovery outcomes with GAP-43, SYN, and Bcl-2/bax were analyzed using Spearman's correlation coefficient. *P* < 0.05 was considered to be a statistically significant difference.

## 3. Results

### 3.1. Effects of an EE on Physical Function

**Figure 3:**
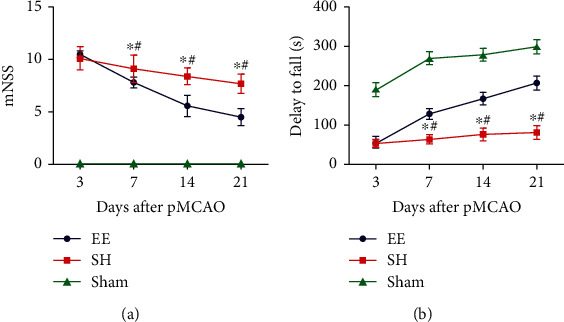
Effect of an EE on limb motor function (mNSS and Rotarod tests). (a) Evaluation of recovery of neurological deficits, using the mNSS test following pMCAO. Mice in the EE group exhibited greater enhancement on days 7, 14, and 21 post pMCAO compared to the SH group on those days. ^∗^*P* ≤ 0.001 vs. sham group. ^#^*P* = 0.007 vs. EE group. (b) Evaluation of the movement ability of mice, using the Rotarod test after pMCAO: the EE group stayed longer on the rotor on days 7, 14, and 21 post pMCAO compared to the SH group. ^∗^*P* ≤ 0.001 vs. sham group. ^#^*P* ≤ 0.001 vs. EE group. Data are expressed as the mean ± SEM (*n* = 8; one-way analysis of variance followed by Fisher's least significant difference post hoc test).

The mNSS test was used to evaluate the recovery of mice from neurological deficits. As shown in Figure 3(a), mNSS test scores were more markedly improved on days 7, 14, and 21 after operation in the EE group compared to the SH group (*P* < 0.05). The Rotarod test was used to evaluate the movement ability of mice, and the results revealed that the movement ability of mice in the EE group was significantly improved compared to the SH group (Figure 3(b), *P* < 0.05).

### 3.2. Western Blot Assay of Bcl-2, Bax, GAP-43, and SYN Proteins

**Figure 4:**
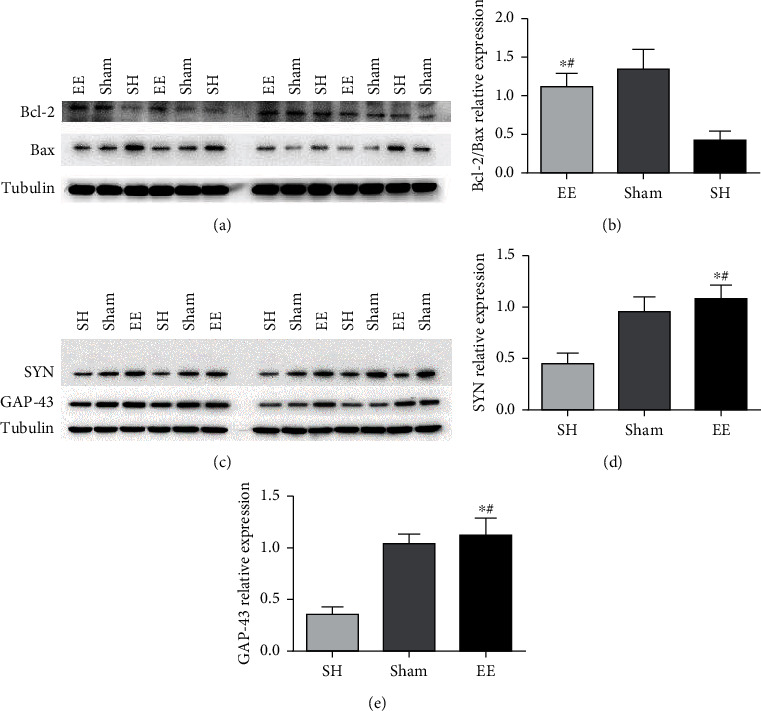
Effects of EE on Bcl-2 and Bax (a–c) and SYN and GAP-43 (d, e) relative expressions in the peri-infarct cortex. (a) Representative western blots of Bcl-2 and Bax. (b) Quantitative analysis of Bcl-2/Bax. ^∗^*P* ≤ 0.001 vs. SH group. ^#^*P* = 0.089 vs. sham group. (c) Representative western blots of SYN and GAP-43. (d) Quantitative analysis of SYN. ^∗^*P* ≤ 0.001 vs. SH group. ^#^*P* = 0.133 vs. sham group. (e) Quantitative analysis of SYN. ^∗^*P* ≤ 0.001 vs. SH group. ^#^*P* = 0.191 vs. sham group. Data are expressed as the mean ± SEM (*n* = 8; one-way analysis of variance followed by Fisher's least significant difference post hoc test). Bax: Bcl-2-associated X protein; Bcl-2: B-cell lymphoma-2; EE: enriched environment; GAP-43: growth-associated protein 43; SH: standard housing; SYN: synaptophysin.

The expression of Bcl-2, Bax, GAP-43, and SYN protein bands in the EE group was significantly greater than in the standard housing group (Figure 4, *P* < 0.05).

### 3.3. Immunofluorescence Assay of Bcl-2, Bax, GAP-43, and SYN

**Figure 5:**
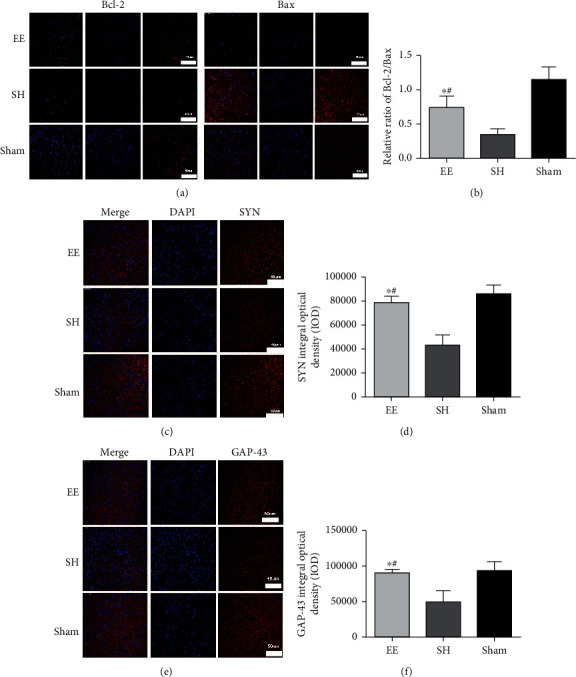
Effects of EE on Bcl-2 and Bax (a–c) and SYN and GAP-43 (d–f) expressions in the peri-infarct cortex. SYN integral optical density (IOD). (a) Representative immunofluorescence images of Bcl-2 and Bax (red) in the EE, SH, and sham groups. Scale bar = 50 *μ*m. (b) Bar graph showing the immunofluorescence quantification of the expression of Bcl-2/Bax among different groups. ^∗^*P* ≤ 0.001 vs. SH group. ^#^*P* = 0.002 vs. sham group. (c) Representative immunofluorescence images of SYN (red) in the EE, SH, and sham groups. Scale bar = 50 *μ*m. (d) Bar graph showing the immunofluorescence quantification of the expression of SYN in the different groups. ^∗^*P* ≤ 0.001 vs. SH group. ^#^*P* = 0.068 vs. sham group. (e) Representative immunofluorescence images of GAP-43 (red) in the EE, SH, and sham groups. Scale bar = 50 *μ*m. (f) Bar graph showing the immunofluorescence quantification of the expression of GAP-43 in the different groups. ^∗^*P* ≤ 0.001 vs. SH group. ^#^*P* = 0.102 vs. sham group. Data are expressed as the mean ± SEM (*n* = 8; one-way analysis of variance followed by Fisher's least significant difference post hoc test). Bax: Bcl-2-associated X protein; Bcl-2: B-cell lymphoma-2; EE: enriched environment; GAP-43: growth-associated protein 43; SH: standard housing; SYN: synaptophysin.

The immunofluorescence assay results revealed that the EE group had a higher expression of Bcl-2/Bax, GAP-43, and SYN compared to the control group of mice (Figure 5, *P* < 0.05), which demonstrated that an EE treatment could upregulate Bcl-2/Bax, GAP-43, and SYN expressions, which all promote neurogenesis and neuroplasticity.

### 3.4. Correlation of Physical Function Outcomes with Bcl-2/Bax, GAP-43, and SYN

**Figure 6:**
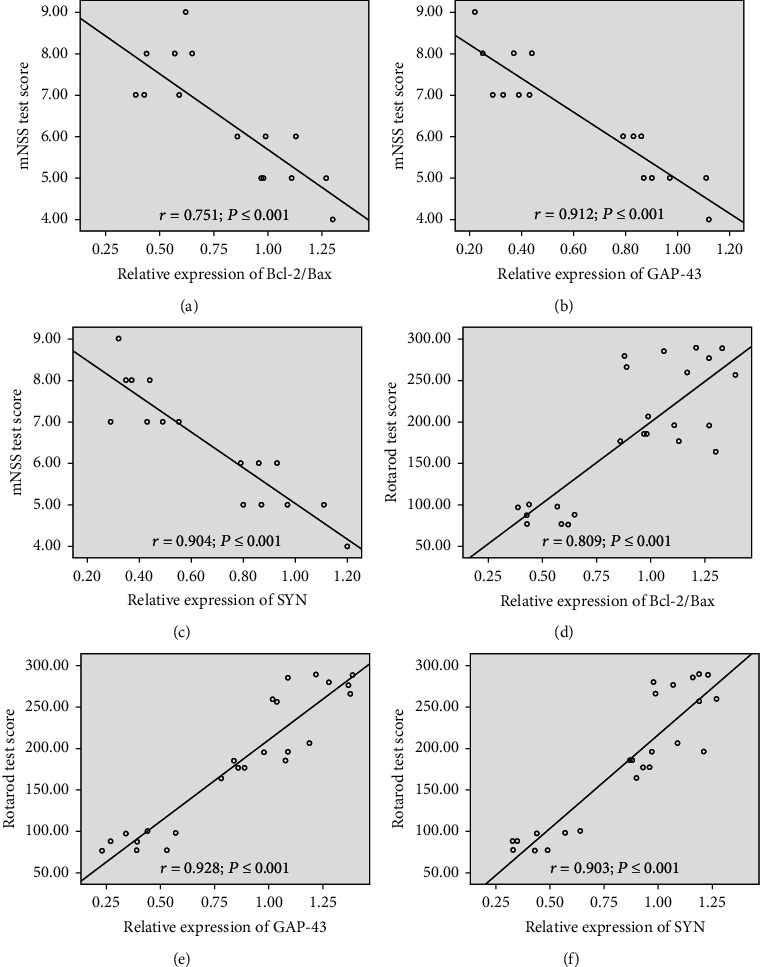
Correlation of physical function outcomes with Bcl-2/Bax, GAP-43, and SYN. The physical function outcomes were positively correlated with the relative expression of Bcl-2/Bax, GAP-43, and SYN, and the correlation between the GAP-43 and SYN is stronger than that of the Bcl-2/Bax. (a) Bcl-2/Bax between mNSS, *r* = 0.751, *P* ≤ 0.001. (b) GAP-43 between mNSS, *r* = 0.912, *P* ≤ 0.001. (c) SYN between mNSS, *r* = 0.904, *P* ≤ 0.001. (d) Bcl-2/Bax between Rotarod, *r* = 0.809, *P* ≤ 0.001. (e) GAP-43 between Rotarod, *r* = 0.928, *P* ≤ 0.001. (f) SYN between mNSS, *r* = 0.904, *P* ≤ 0.001. *r* = Spearman's correlation coefficient. *P* ≤ 0.001 was considered to indicate a significant correlation. Bcl-2: B-cell lymphoma-2 (Bcl-2); Bax: BCL-2-associated X protein; GAP-43: growth-associated protein 43; SYN: synaptophysin.

In order to assess a possible correlation between physical function outcomes and Bcl-2/Bax, GAP-43, SYN, and assess the role of neuronal apoptosis and synaptic remodeling in the improvement of limb function. We compared the physical function outcomes (the mNSS and the Rotarod test scores) to the relative expression of Bcl-2/Bax, GAP-43, and SYN by using Spearman's correlation coefficients (*r*). Correlations between the physical function outcomes and relative expression of Bcl-2/Bax, GAP-43, and SYN are shown in Figure 6. The physical function outcomes were positively correlated with the relative expression of Bcl-2/Bax, GAP-43, and SYN, and the correlation between the GAP-43 and SYN is stronger than that of the Bcl-2/Bax (Figures 6(a)–6(f)).

## 4. Discussion

Benign stimulation by the EE can improve the functional and structural remodeling of the injured brain [16, 17]. Many studies have reported that EE stimulation can improve limb motor dysfunction induced by ischemic stroke [18, 19]. It is widely accepted that the potential internal mechanisms include enhanced synaptic remodeling [20], angiogenesis [21], and neurogenesis [22, 23]. However, only a few studies have focused on synaptic remodeling and neuronal apoptosis simultaneously. The present study explored the effects of an EE on limb motor function recovery and the internal mechanism involved in neuroprotection poststroke.

It was found that EE treatment starting 3 days after pMCAO improved the limb motor function recovery 21-day poststroke and that this recovery may be because of improved neuroprotection induced by the increased expression of GAP-43 and SYN, as well as the ratio of Bcl-2/Bax. In this study, a pMCAO model was employed, which results in irreversible damage to certain brain regions [24]. This model ensures consistency and repeatability of experimental results after uniform functional disorder poststroke [25].

Synaptogenesis is considered essential for the recovery of limb functions after stroke. Synaptic remodeling includes the prolongation of axons and an increase in synaptic-associated proteins. GAP-43 is a unique growth-related protein of nerve tissue, which is widely distributed in neurons, regenerated Schwann cells and glial cells, and especially in the ends of growing neurons [26]. Therefore, GAP-43 is considered to be a sensitive marker of axon sprouting and prolongation in an after-stroke model [27]. SYN is an important marker associated with presynaptic membrane genesis, which is closely related to the synaptic structure and its functions [28]. A previous study has shown that SYN is an important marker of synaptic reestablishment after cerebral ischemic [29]. The experimental data revealed that GAP-43 and SYN expressions were obviously upregulated in mice exposed to EE, suggesting that EE can significantly promote synaptic remodeling in the peri-infarct area.

Neuronal apoptosis is a complex pathophysiological process regulated by a variety of factors and is one of the main causes of neuronal death after cerebral ischemia [30, 31]. Sufficient neuronal protection can prevent cell death or apoptosis after brain injury. Studies have shown that Bcl-2 and Bax are key proteins regulating apoptosis [32, 33]. Both passive exercise and preischemic active exercise can inhibit apoptosis and reduce neuronal loss in the peri-infarct area by upregulating the ratio of Bcl-2/Bax. In our study, it was found that the Bcl-2 and Bax were upregulated and downregulated by an EE, respectively, suggesting that EE has a protective effect on neurons in the peri-infarct area by reducing neuronal apoptosis.

Previous studies have shown that the mechanisms involved in EE-enhanced functional recovery after ischemic stroke are diverse but still remain unclear. Which mechanism plays a leading role deserves further study, which is helpful to further understand the internal mechanism of EE. Our present study suggests that EE plays a synergistic role in promoting synaptic remodeling and in reducing neuronal apoptosis poststroke simultaneously. Through correlation analysis, we further found that comparing with neuronal protection, synaptic remodeling plays a dominant role in limb functional recovery. This finding is helpful to further study the internal relationship of multiple mechanisms of EE in neural plasticity.

A limitation of the study was that we only observed several representative key indicators of synaptic remodeling and neuronal apoptosis, without an in-depth study of the potential mechanisms involved. In the near future, we intend to study the molecular pathways underlying the neuroprotective effect of EE on cerebral ischemia.

In conclusion, our present study found that EE can promote the process of the improvement of limb dysfunction induced by ischemic stroke, and this behavior restoration may, via promoting neuroprotection in the ischemic penumbra, be dependent on the regulation of the expression of GAP-43, SYN, and Bcl-2/Bax. We also found that compared with neuronal protection, synaptic remodeling plays a more important role in limb function recovery.

## Data Availability

Datasets analyzed during the current study are available from the corresponding author on reasonable request.
